# Achieving the HIV Prevention Impact of Voluntary Medical Male Circumcision: Lessons and Challenges for Managing Programs

**DOI:** 10.1371/journal.pmed.1001641

**Published:** 2014-05-06

**Authors:** Sema K. Sgaier, Jason B. Reed, Anne Thomas, Emmanuel Njeuhmeli

**Affiliations:** 1 Integrated Delivery, Global Development Program, Bill & Melinda Gates Foundation, Seattle, Washington, United States of America; 2 Department of Global Health, University of Washington, Seattle, United States of America; 3 Office of the U.S. Global AIDS Coordinator, Washington (DC), United States of America; 4 Naval Health Research Center, US Department of Defense, San Diego, California, United States of America; 5 United States Agency for International Development, Washington (DC), United States of America

## Abstract

Voluntary medical male circumcision (VMMC) is capable of reducing the risk of sexual transmission of HIV from females to males by approximately 60%. In 2007, the WHO and the Joint United Nations Programme on HIV/AIDS (UNAIDS) recommended making VMMC part of a comprehensive HIV prevention package in countries with a generalized HIV epidemic and low rates of male circumcision. Modeling studies undertaken in 2009–2011 estimated that circumcising 80% of adult males in 14 priority countries in Eastern and Southern Africa within five years, and sustaining coverage levels thereafter, could avert 3.4 million HIV infections within 15 years and save US$16.5 billion in treatment costs. In response, WHO/UNAIDS launched the Joint Strategic Action Framework for accelerating the scale-up of VMMC for HIV prevention in Southern and Eastern Africa, calling for 80% coverage of adult male circumcision by 2016. While VMMC programs have grown dramatically since inception, they appear unlikely to reach this goal. This review provides an overview of findings from the PLOS Collection “Voluntary Medical Male Circumcision for HIV Prevention: Improving Quality, Efficiency, Cost Effectiveness, and Demand for Services during an Accelerated Scale-up.” The use of devices for VMMC is also explored. We propose emphasizing management solutions to help VMMC programs in the priority countries achieve the desired impact of averting the greatest possible number of HIV infections. Our recommendations include advocating for prioritization and funding of VMMC, increasing strategic targeting to achieve the goal of reducing HIV incidence, focusing on programmatic efficiency, exploring the role of new technologies, rethinking demand creation, strengthening data use for decision-making, improving governments' program management capacity, strategizing for sustainability, and maintaining a flexible scale-up strategy informed by a strong monitoring, learning, and evaluation platform.

## Abstract 2

In this Collection Review, Sema Sgaier and colleagues highlight the key points from the PLOS Volunteer Medical Male Circumcision Collection and give some recommendations on the way forward.

*Please see later in the article for the Editors' Summary*

**Summary Points**

- Large-scale implementation of voluntary medical male circumcision (VMMC) in 14 priority countries of Eastern and Southern Africa has the potential to significantly reduce heterosexual transmission of HIV to males, saving lives, averting suffering, and avoiding health care costs.
- Resource and capacity constraints pose a serious challenge to the ability of the priority countries to reach their goals for VMMC scale-up.
- The 13 papers in this collection examine issues of service quality, demand creation, cost, and efficiency faced by governments, donors, and programs.
- Systematic, evidence-based management of programs and a dynamic culture of learning are proposed to help meet the challenges of VMMC scale-up.
- Recommendations include greater prioritization and funding of VMMC, strategic targeting and demand creation, a focus on programmatic efficiencies, and exploration of new technologies.
- Further recommendations are for strengthened data use, improving governments' program management capacity, strategizing for sustainability, and maintaining a flexible scale-up strategy.

## Introduction

**Figure 1:**
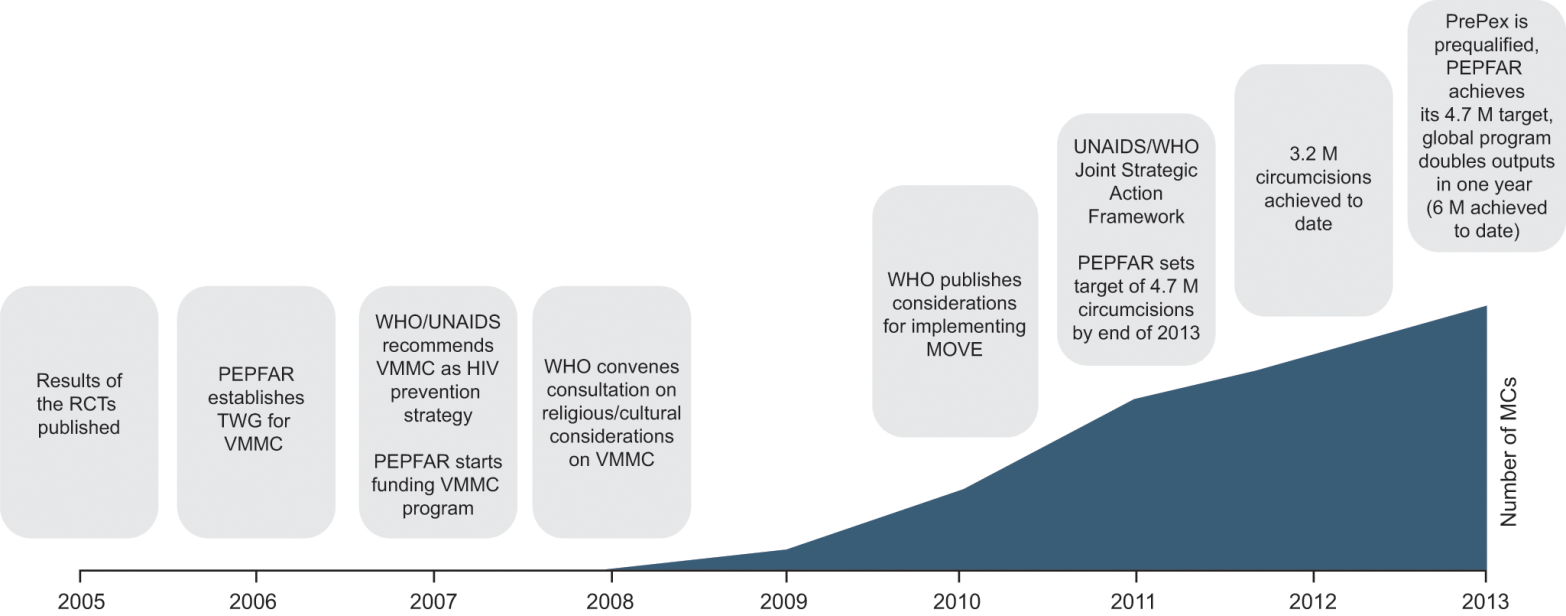
Timeline and key milestones of the voluntary medical male circumcision program in 14 priority countries. 6 million circumcisions listed in 2013 is an estimate by PEPFAR and the Bill & Melinda Gates Foundation. RCTs, randomized controlled trials; TWG, technical working group; TAG, technical advisory group; MOVE, Models for Optimizing the Volume and Efficiency of MC services.

Voluntary medical male circumcision (VMMC) has been shown to be effective in reducing the sexual transmission of HIV from women to men (Figure 1) [1]–[4]. Fourteen countries in Eastern and Southern Africa with high HIV prevalence and low levels of male circumcision (MC) are following the recommendations of the World Health Organization (WHO) and the Joint United Nations Programme on HIV/AIDS (UNAIDS) to expand VMMC services as an HIV prevention strategy [5],[6]. These countries are Botswana, Ethiopia, Kenya, Lesotho, Malawi, Mozambique, Namibia, Rwanda, South Africa, Swaziland, Uganda, the United Republic of Tanzania, Zambia, and Zimbabwe. Modeling studies done in 2009–2011 showed that in these 14 priority countries, achieving 80% circumcision prevalence among males aged 15–49 within five years (“catch-up”), and maintaining this coverage rate in subsequent years (“sustainability”), could avert 3.4 million new HIV infections within 15 years and generate treatment and care savings of US$16.5 billion [7],[8]. VMMC is a highly cost-effective HIV prevention strategy for both generalized and high-prevalence HIV epidemics [7]. It differs from most other prevention methods (e.g., pre-exposure prophylaxis, sexual behavior change, or condom use) in that it only requires a one-time action in order to provide continuous benefits. VMMC has political support at both the global and national levels. Most of the 14 priority countries have developed strategic plans and established infrastructure to implement the plans and scale up VMMC interventions. (These efforts are referred to collectively in this paper as “the VMMC program” or “the program.”)

**Figure 2:**
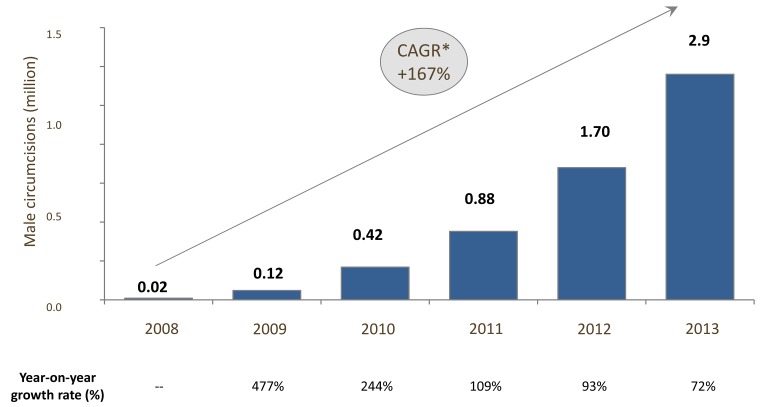
Scale-up of voluntary medical male circumcision program and coverage in 14 priority countries, aggregate, 2008–2013. Number of circumcisions completed each year in millions. Source of 2008–2012 data is the WHO 2012 VMMC report [38]. 2013 numbers have been estimated using data from PEPFAR and the Bill & Melinda Gates Foundation. *CAGR, compound annual growth rate, calculated based on the average proportional growth each year. CAGR (t_0_,t_n_)  =  (V(t_n)_/V(t_0_))^1/(tn − to)^ −1, where V(t_0_) is the start value and V(t_n_) is the finish value and t_n_ − t_0_ is the number of years.

**Figure 3:**
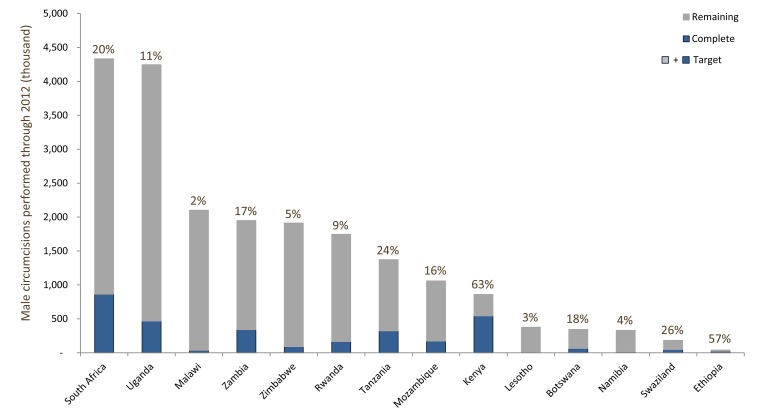
Scale-up of voluntary medical male circumcision program and coverage in 14 priority countries, 2008–2012. Totals reflect progress through 2012. Percentage figures represent the achieved proportion of the target of 80% coverage among males ages 15–49, but totals include circumcisions done for all age groups, regardless of the age-range target. Data obtained from WHO 2012 VMMC report [38].

**Figure 4:**
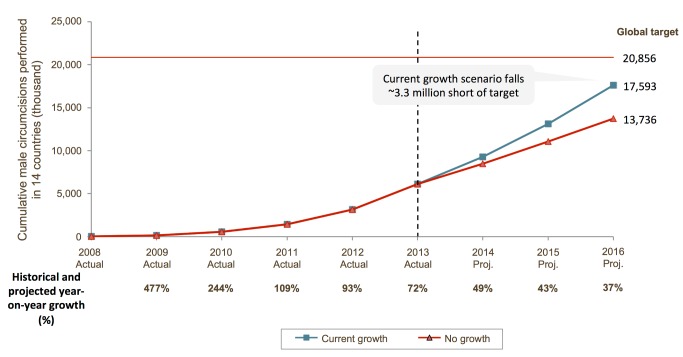
Scale-up of voluntary medical male circumcision program and coverage in 14 priority countries: growth scenarios, 2008−2016. Source of 2008–2012 data is the WHO 2012 VMMC report [38]; 2013 figures are estimates, and 2014–2016 figures are projections. In the “no growth” scenario, the program continues to perform the same numbers of circumcisions each year as in 2013. In the “current growth” scenario, the program continues the trend of historical growth rate.

Nevertheless, the VMMC strategy faces challenges at multiple levels. The goals set out in the Joint Strategic Action Framework (JSAF) are highly ambitious: to circumcise 20.2 million men in five years (2012–2016) across 14 African countries [6]. Furthermore, the choice to be circumcised involves deep-seated values, beliefs, and motivational factors that vary with ethnic, religious, and cultural identities, and must be addressed effectively to generate demand for circumcision. While at a broad level the program is increasing its outputs each year (Figures 2 and 3), the current growth rate of the program is not sufficient to reach the JSAF's goal of 80% coverage by 2016 (Figure 4).

This collection of research and review articles, “Voluntary Medical Male Circumcision for HIV Prevention: Improving Quality, Efficiency, Cost Effectiveness, and Demand for Services during an Accelerated Scale-up,” presents new research that underscores both the challenges and the opportunities for the accelerated scale-up of VMMC (Box 1). This review article synthesizes and briefly describes the findings, which focus on four key areas of the VMMC program: (1) quality of services, (2) demand creation, (3) cost, and (4) efficiencies for service delivery. Building on these findings, we propose a set of priority focus areas for the global VMMC program and for the governments of the priority countries, and we highlight new areas of opportunity to accelerate scale-up.

**Box 1. Scope of the Collection**

The PLOS Collection “Voluntary Medical Male Circumcision for HIV Prevention: Improving Quality, Efficiency, Cost Effectiveness, and Demand for Services during an Accelerated Scale-up” includes 13 research papers and an overview that highlight key findings from several countries that have moved to high-volume VMMC services, explore challenges faced, and offer recommendations for programs as they scale up VMMC services. It focuses on the following:

- Quality and efficiency of VMMC services – results of the Systematic Monitoring of the Voluntary Medical Male Circumcision Scale Up (SYMMACS) facility-based survey conducted in Kenya, South Africa, Tanzania, and Zimbabwe
- Costing and Impact – findings from three studies by PEPFAR through the USAID funded Health Policy Project of interest to decision-makers determining allocation of HIV prevention resources
- Demand Creation – studies from Kenya, Zimbabwe, and Tanzania highlight importance of tailoring demand-creation interventions and service delivery models to specific age group of clients

### Quality of VMMC Services

Ensuring safe and efficient execution of the VMMC procedure and high-quality pre- and post-procedure services, even as programs expand rapidly, is essential in order to realize the full impact of VMMC, reduce program costs, and increase and sustain demand for the service. While most previous quality assessments focused on adverse events in clients [9], two papers in this collection examine facility preparedness for providing quality surgical services using data across two years of program scale-up. Jennings and colleagues undertook a comparative assessment of facility preparedness and surgery in Kenya, South Africa, Tanzania, and Zimbabwe through direct observation [10]. Results for facility preparedness were mixed: in some settings, improvements were seen over time, while in others, early problems that went uncorrected prior to expansion were amplified system-wide at scale. Rech and colleagues explored in further detail the implications of scale-up in South Africa [11]. They assessed readiness to provide quality services and quality of surgical care in 2011 and 2012. Rapid scale-up in South Africa led to human resources being stretched too thinly, with negative effects on quality overall. These studies, and a further study of surgical efficiencies and quality of surgical technique in VMMC in Kenya, South Africa, Tanzania, and Zimbabwe [12], remind us that while it is possible to maintain high standards of surgical care during rapid scale-up (Zimbabwe is a good example), there is always a need to manage adverse events by proactively following up with clients and facilitating postoperative services. As programs expand, it is also necessary to improve initial and refresher training for providers on all aspects of the VMMC service package, strengthen ongoing quality and performance assessments, reinforce supervision and mentoring, and create functional systems to continuously assess and improve service quality.

### Demand Creation

There is general agreement among the authors about the need to foster greater demand among men for VMMC in order to achieve the desired reduction in HIV incidence [6]. Although most programs aim to reach men aged 15–49, those over 25 are underrepresented thus far in the scale-up [13]. Two studies have explored barriers to and facilitators of demand [14], and three papers in this collection specifically address men aged 25 and above, a group for whom the reasons for low demand are not well understood, and whose contribution to HIV prevalence is currently being evaluated by new modeling work.

Macintyre and colleagues examined barriers to and facilitators of demand in Turkana County, Kenya, a traditionally non-circumcising community [15]. Older married men did not consider themselves at risk of acquiring HIV, and they echoed the findings of other literature on acceptability in viewing circumcision as more appropriate for younger men, whom they perceived as being at higher risk of HIV [16]–[20]. The authors argue that promoting circumcision as a modern, biomedical procedure rather than as a cultural practice (which may be associated with other tribal groups or young men's rites of passage) will be an important demand-creation message in this particular setting.

In Zimbabwe, Hatzold and colleagues conducted quantitative and qualitative studies to explore barriers to and motivating factors for VMMC and to assess the use of existing VMMC communication channels to promote VMMC [21]. A large majority of the respondents had heard about VMMC, mainly through radio. Prevention of HIV was the most cited motivator for circumcision, but men were also motivated by prospects for improved hygiene. Non-HIV-related benefits could be part of the messaging for demand-creation activities. Only 11% of all the men surveyed had been circumcised, and among male respondents expressing an unwillingness to become circumcised, fear of pain was the most frequently cited reason. The paper does not directly identify the reasons why men who expressed a willingness to undergo VMMC had not yet done so, and further research on this would be useful. However, three primary predictors of VMMC uptake were identified: self-efficacy (the belief that one can make the decision oneself to be circumcised), social support from friends, and availability of VMMC services.

The study of the modality and intensity of service delivery in Tanzania and Zimbabwe by Adamu and colleagues [22] shows that campaigns conducted during school holidays lead to higher attendance by younger clients, an effect enhanced in Tanzania by the cultural preference for circumcision at a younger age [23]. This underscores the importance of considering cultural preferences and service delivery timing when designing demand-creation approaches.

### Costs

While modeling showed that scaling up VMMC to 80% coverage among men aged 15–49 in the 14 priority countries could result in savings of US$16.5 billion in treatment costs, the resources required to achieve this are substantial—an estimated US$2 billion [7],[8],[24]. Three papers in this collection undertake unit cost analyses to identify the cost drivers and explore where efficiency gains are possible. Bollinger and colleagues (data from Kenya, Namibia, South Africa, Tanzania, Uganda, and Zambia) and Menon and colleagues (data from three regions of Tanzania) use programmatic and financial data (including direct and indirect costs) from VMMC facilities to determine the unit cost of a surgical VMMC [24],[25]. Both studies illustrate that the two largest cost drivers are personnel (36% of costs) and commodities (28%), with ranges varying by country and model of service delivery.

An economies-of-scale analysis suggests that further cost reductions will be seen as the supply side of the program plateaus [24]. These studies highlight the possibility of further efficiency gains in the personnel and consumables components of the program by maximizing efficiencies in human and material resources and technology.

Three independent modeling studies, including one in this issue, using different starting assumptions, have forecast little if any cost savings with the initial use of circumcision devices such as PrePex and Shang Ring [26]–[28]. Njeuhmeli and colleagues note that the single greatest determinant of unit cost was site utilization, with underutilization doubling the per-procedure price, regardless of whether circumcision was device-based or surgical [28]. Device-based circumcision has yet to be provided as a routine procedure under efficiency conditions, as surgical circumcision is currently provided. If device-based service models function closer to maximum capacity, decreased unit costs could be realized. However, there will always be a need for national programs to provide surgical circumcision for men who are not eligible for devices or who prefer surgery.

A major limitation of the cost studies in this collection is that they do not include the costs of demand creation nor analyze how increased spending on demand creation might affect the actual demand for services. This is partly because of limitations in the available data. In future studies, it will be important to address this knowledge gap.

### Efficiencies

Several papers in this collection explore improvements in the efficiency of service delivery, with specific emphasis on efficiencies around the surgical procedure, using data from Kenya, South Africa, Tanzania, and Zimbabwe [9]. None of the four countries surveyed had adopted all six elements of surgical efficiency tracked by the data, which were (1) use of multiple surgical beds, (2) use of pre-bundled surgical supply kits, (3) task-shifting, (4) task-sharing, (5) use of forceps-guided surgical method, and (6) use of electrocautery. The use of the forceps-guided surgical method was the only element adopted by all countries. Rech and colleagues found that innovative human resource models such as the sharing of tasks among a coordinated team of clinicians, as well as electrocautery and the use of multiple beds, reduced the overall procedure time and increased the number of clients served by the primary surgeon in a given period of time [12]. An encouraging finding was that the quality of surgical technique was not compromised by reducing the amount of time the primary provider spent with each client, meaning that quality was maintained in high-service-volume settings. In fact, in South Africa, reduced operating time was associated with higher surgical quality, which the authors suggest might be the result of experienced providers being more skilled and working more efficiently.

While these papers focus on efficiencies that could be gained at the site level, an assessment of efficiency gains at the macro level of overall program delivery is also needed, as discussed below.

Mavhu and colleagues found that providers held positive attitudes toward the six recommended elements of surgical efficiency in countries where national policies were supportive [29]. This study highlights the value of consulting with those who will implement policies at the service-delivery level. The finding that national policy bodies in South Africa and Zimbabwe did not adopt task-shifting—even though providers supported it—is significant, because task-shifting could alleviate the human resources constraint that is a major challenge to the scale-up of VMMC in these countries.

Perry and colleagues identified factors associated with provider burnout (physical or emotional exhaustion as a result of prolonged stress or frustration) and explored a possible relationship between burnout and job satisfaction [30]. In 2012, more than three-fourths of providers surveyed in Kenya, South Africa, Tanzania, and Zimbabwe reported that providing VMMC was personally fulfilling. Providers' median work span in VMMC was longest in Kenya (31 months), but the proportion of providers who reported that they were starting to experience burnout was much higher there (67%) than in the other three countries. However, the study failed to identify any correlates associated with burnout in Kenya.

## Discussion

The VMMC program is an ambitious public health intervention. While it is estimated that close to 6 million circumcisions had been completed by the end of 2013, against a goal of 20.2 million by 2016, this progress should be viewed within the context of the recent and rapidly developing understanding of the importance of VMMC as an HIV prevention intervention (Figure 1). Less than three years after a consensus was reached to recommend VMMC as an additional HIV prevention intervention in countries with high HIV prevalence and low MC rates [6], 11 of the 14 priority countries had approved national VMMC strategies and operational plans. The program has been scaling up in many countries since then, with the result that the number of circumcisions performed each year, which in 2008 had been only 0.02 million, grew to an estimate of at least 2.8 million in 2013, representing a compound annual growth rate of 166% (Figure 2).

Despite the rapid implementation and scale-up of VMMC programs and the doubling of the cumulative total VMMC procedures in the past year (from 3.2 million by the end of 2012 to an estimated 5.8–6 million by the end of 2013), progress at the country level has varied widely (Figure 3) and the year-on-year rate of growth in the number of VMMCs performed is declining (Figures 2 and 4). This is due to a combination of factors: the JSAF goal of reaching 80% of uncircumcised men by 2016 did not fully take into account country-specific constraints that have tempered the pace of scale-up, lack of sufficient demand, and insufficient funding from a broad base of international donors (the United States President's Emergency Plan for AIDS Relief [PEPFAR] has funded more than 80% of circumcisions to date). Modeling suggests that even if the current growth rate is maintained and adequate funds are forthcoming, the number of VMMCs completed by 2016 would fall about 3 million short of the JSAF goal of 20.2 million (Figure 4).

This collection provides an opportunity to reflect on ways to maximize the potential of the VMMC program during the remainder of the accelerated scale-up period (until 2016) (Box 2). This means prioritizing strategies that can improve programmatic impact and efficiency in order to avert the greatest possible number of HIV infections through VMMC within realistic cost and capacity constraints, as well as taking into account funds available for VMMC.

**Box 2. Key Points from the Collection**

- **Quality of services:** It is possible to maintain and even improve service quality, especially surgical performance, as VMMC is scaled up, but improved provider training is needed to strengthen quality of pre- and postoperative care and infection control.
- **Costs:** Personnel and consumables are the largest cost drivers, but costs may be reduced as programs scale up and economies of scale are achieved, as well as by improving service efficiency. Underutilization of service capacity increases unit costs more than any other variable, highlighting the importance of predictable demand and nimble service platforms so that sites are consistently performing as close to capacity as possible. Responsible public-sector pricing strategies for devices have the potential to reduce overall unit costs, and further discounts should be negotiated as procurement volumes increase.
- **Demand creation:** Messaging must be tailored to different age groups and to the cultural norms of different communities. Men aged 25 and above are less motivated to undergo VMMC. Studies suggest that we need to go beyond simple HIV messaging and present VMMC in terms of hygiene, appearance, attractiveness to partners, peer group norms, and modernity.
- **Efficiencies:** Adoption of various elements of surgical efficiency is variable between countries studied. Task-sharing, sharing, bundling of surgical instruments, and electrocautery are associated with surgical efficiency outcomes, and surgical quality need not be compromised by measures to reduce operating time.

**Figure 5:**
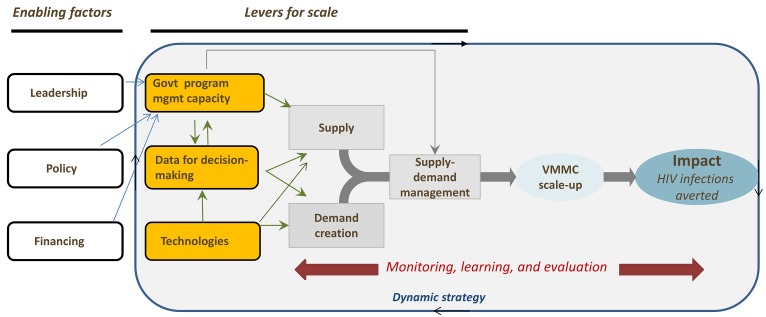
Enabling factors and levers to achieve scale and impact for the voluntary medical male circumcision program. Strong enabling factors of leadership, policy, and financing are needed to accelerate and maximize the impact of scale-up of the VMMC program. The levers for scale—government management capacity, use of data for decision-making, and technologies—are needed to match supply and demand.

We believe it is essential to take a systematic approach to improving the design, implementation, and evaluation of the program. Several enabling factors and levers may accelerate and maximize the impact of scale-up (Figure 5). These enabling factors—leadership, policy, and to a certain extent funding—are largely in place both globally and in VMMC priority countries. Increased funding for VMMC is needed to meet the growing targets and outputs of the program. Given that PEPFAR is the predominant donor, getting other donors and governments to contribute is essential. In addition, the levers for scale identified in Figure 5 (government program management capacity, use of data for decision-making, and technologies) require further strengthening in order to match supply and demand. This challenge may be met by incorporating principles of managing large-scale enterprises such as standardization of service delivery and training, market segmentation, and data-driven management [31]. Building on the findings presented in this collection, we propose below a set of actions for the VMMC community.

### Action Recommendations

#### Continue advocating for prioritization and funding for VMMC

The program faces increasing competition for declining funding for HIV prevention and treatment. The funding need for VMMC remains significant, and continued evidence-based advocacy is necessary to secure funds for accelerated scale-up from a broad base of donors. One way to do this is to evaluate the population-level impact of those VMMC programs that have already been scaled up. Another is to continue to draw comparisons between VMMC and other HIV prevention programs, highlighting specifically that if VMMC coverage reaches the JSAF goal of 80%, it will prove the most cost-effective and cost-saving HIV prevention intervention in Eastern and Southern Africa. In addition, it does not require sustained adherence, and there is evidence that referrals made from the VMMC program increase HIV-positive males' access to treatment [32].

#### Increase program efficiency by identifying and prioritizing those most at risk of acquiring HIV

Circumcisions performed on those males most at risk of acquiring HIV will have the greatest epidemiological impact in terms of HIV infections averted [7]. There is an opportunity to strategically prioritize subpopulations (for example, by age and geography) to maximize the program's impact and efficiency within the required time period, while also ensuring that VMMC is available to all males who want it. Given what is known about the relatively low uptake of VMMC among men aged 25 and above [14],[23], we also recommend more modeling to ascertain the relative contribution of this subgroup to overall infections averted in the short and long terms.

#### Focus on program efficiency and quality at all levels, and on matching supply with demand

While a large portion of this collection focuses on surgical efficiency or quality [9]–[12], the bigger challenge is overall programmatic efficiency. The challenge of scale-up can be approached as a management challenge that requires addressing each element of the delivery value chain (the specific activities that deliver the end product to the user), using time and resources appropriately, and matching supply with demand for VMMC services while working to increase both. Human resource constraints must be addressed in some places (e.g., through the sharing of clinical tasks [33]), while mechanisms to monitor the quality and safety of the rapidly expanding programs must also be enhanced [11],[12]. Advocacy for including surgical circumcision within the scope of practice for nurses (task-shifting) should be sustained in those countries that have not yet adopted it. Supply of VMMC services must be calibrated to meet demand and located in areas where it will reach the prioritized populations.

#### Explore the role technologies, especially devices, can play in accelerating scale-up

The cost of circumcision devices and other supply chain costs must be brought down considerably if devices are to reduce overall program costs. This will require advocacy as well as negotiation with manufacturers and suppliers in tandem with demand-creation activities. We recommend further study to ascertain whether devices make circumcision more attractive to men and to understand whether devices could assist with balancing supply and demand to help achieve needed programmatic efficiencies. It is also important to tailor demand-creation activities for devices in order to reach those who may already be aware of circumcision's benefits but who have avoided conventional surgical methods.

#### Rethink demand creation through market segmentation and insights from other disciplines

More needs to be done not only to stimulate demand for VMMC [15],[21],[22], but also to normalize it, or at least to better forecast the fluctuations in demand that are apparent in many places [15]. Barriers and facilitators to uptake of services must be understood, with male populations properly segmented demographically to make best use of limited resources. To date little research has looked at the male population as a market of consumers of an intervention with multiple benefits. A market research approach, along with insights from diverse fields such as behavioral economics and anthropology, can provide new tools to inform the development of new approaches. More funding should be allocated to systematically evaluate the effectiveness of the many approaches to creating and mobilizing demand. Those that show positive results should be taken to scale.

#### Gather and use standardized, high-quality data for program management and decision-making

A broad range of data should be collected and reported on a frequent and regular basis and used by program managers at all levels to analyze program constraints and manage supply and demand [34]. Examples of such data include the performance of a site (i.e., the number of VMMCs performed) against its functional capacity and projected demand; the demand-creation channels that bring individuals to specific VMMC sites; daily outputs of community members engaged to generate demand; and information on seasonal fluctuations in demand [35].

We recommend that systems and tools be developed to empower and enable managers to use data for day-to-day decision-making and to adjust services in real time. Useful lessons may be learned from other programs (e.g., the Avahan India HIV prevention program), including the use of micro-planning [36]. There is a related need for continual analysis of cost drivers to see where efficiency gains are possible [24]. However, cost analysis should look at the overall program—not just the cost of individual procedures—and should include management, the cost of demand-creation activities, and the opportunity cost of underutilized services sites.

#### Strengthen government capacity to manage and coordinate programs

We recommend exploring ways to support the program management capacity of national and subnational governments to coordinate multiple donors and implementers [37], manage competition for limited human resources and health infrastructure, and avoid duplication of efforts. Governments must also lead in strategic planning, including target-setting, geographic prioritization, quality assurance, and monitoring and evaluation. Related to this is the need to support government capacity for streamlined data collection using tracking systems that are standardized across implementers.

#### Strategize for the sustainability phase of the program

It is important to begin strategizing for the sustainability phase that should follow the present “catch-up” activities. It will take time to determine the best approach to sustaining high MC prevalence in each country, develop global and national frameworks, secure resources, and implement long-term programs. Since the cohorts prioritized in the sustainability phase will likely be some combination of uncircumcised boys (aged 10−14 years) and infants (aged 0−60 days), it will also be important to explore how best to reach young adolescents and parents of infants, taking into account impact, cost, the feasibility of scale-up, cultural acceptability, and other factors.

#### Keep strategy dynamic, informed by a strong monitoring, evaluation, and learning platform

Our final recommendation is that ministries of health, donors, program planners, and implementers strive to maintain a dynamic strategy and evidence-based programs that are agile and able to correct their course when necessary. This is crucial because of the constantly developing understanding of the complexities and challenges of managing VMMC supply and demand, dealing with new technologies, managing costs, and improving efficiency and quality. For this reason too, investment in disseminating and incorporating lessons learned, informed by monitoring and evaluation data, should also be prioritized (Box 3).

**Box 3. What Needs to Be Done to Accelerate Scale-up and Impact of the VMMC Program?**

- Continue advocating for prioritization and funding for VMMC.
- Increase program efficiency by identifying and prioritizing those most at risk of acquiring HIV.
- Focus on program efficiency and quality at all levels, and on matching supply with demand.
- Explore the role that technologies, especially devices, can play in accelerating scale-up.
- Rethink demand creation through market segmentation and insights from other disciplines.
- Gather and use standardized, high-quality data for program management and decision-making.
- Strengthen government capacity to manage and coordinate programs.
- Strategize for the sustainability phase of the program.
- Keep strategy dynamic, informed by a strong monitoring, evaluation, and learning platform.

### Conclusion

Large-scale implementation of VMMC has the potential to significantly reduce the incidence of HIV infection among men having heterosexual sex. In the 14 priority countries of Eastern and Southern Africa, the prospective gains through lives saved, suffering averted, and health care costs avoided make VMMC programs a crucial intervention in the struggle against HIV and AIDS. This realization is reflected in the considerable progress already made in scaling up VMMC programs; but resource and capacity constraints pose a serious challenge to the ability of the priority countries to reach the goal set out in the JSAF.

This challenge, readily acknowledged by the countries themselves, is also reflected in a growing body of research that examines the complexity of designing, implementing, scaling up, and evaluating VMMC programs. We believe that many of these challenges can be addressed through systematic, evidence-based management of the programs and a dynamic culture of learning. The papers in this collection add further to our understanding of issues of cost, demand creation, efficiency, and service quality. They represent a significant contribution to our knowledge and help point the way toward the achievement of the VMMC program's ambitious goals.

## Abbreviations

JSAF: Joint Strategic Action Framework
MC: male circumcision
PEPFAR: United States President's Emergency Plan for AIDS Relief
UNAIDS: Joint United Nations Programme on HIV/AIDS
VMMC: voluntary medical male circumcision

## References

[1] Bailey RC, Moses S, Corette BP, Agot K, Maclean I, et al (2007) Male circumcision for HIV prevention in young men in Kisumu, Kenya: a randomized controlled trial. Lancet 369: 643–656. 17321310 10.1016/S0140-6736(07)60312-2

[2] Auvert B, Taljaard D, Lagarde E, Sobngwi-Tambekou J, Sitta R, et al (2005) Randomized, controlled intervention trial of male circumcision for reduction of HIV infection risk: the ANRS 1265 Trial. PLoS Med 2: e298. 16231970 10.1371/journal.pmed.0020298 PMC1262556

[3] Gray RH, Kigozi G, Serwadda D, Makumbi F, Watya S, et al (2007) Male circumcision for HIV prevention in men in Rakai, Uganda: a randomised trial. Lancet 369: 657–666. 17321311 10.1016/S0140-6736(07)60313-4

[4] Weiss HA, Quiqley MA, Hayes RJ (2000) Male circumcision and risk of HIV infection in sub-Saharan Africa: a systematic review and meta-analysis. AIDS 14: 2361–2370. 11089625 10.1097/00002030-200010200-00018

[5] WHO, UNAIDS (2007) New data on male circumcision and HIV prevention: Policy and programme implications. WHO/UNAIDS Technical Consultation on Male Circumcision and HIV Prevention: Research Implications for Policy and Programming. Geneva: WHO.

[6] WHO (2011) Joint Strategic Action Framework to Accelerate the Scale-Up of Voluntary Medical Male Circumcision for HIV Prevention in Eastern and Southern Africa, 2012-2016. Geneva: WHO.

[7] Njeuhmeli E, Forsythe S, Reed J, Opuni M, Bollinger L, et al (2011) Voluntary medical male circumcision: modeling the impact and cost of expanding male circumcision for HIV prevention in eastern and southern Africa. PLoS Med 8: e1001132. 22140367 10.1371/journal.pmed.1001132 PMC3226464

[8] Hankins C, Forsythe S, Njeuhmeli E (2011) Voluntary medical male circumcision: an introduction to the cost, impact, and challenges of accelerated scaling up. PLoS Med 8: e1001127. 22140362 10.1371/journal.pmed.1001127 PMC3226452

[9] Herman-Roloff A, Bailey R, Agot K, Ndinya-Achola J (2010) Medical male circumcision for HIV prevention in Kenya: a study of service provision and adverse events [abstract]. XVIII International AIDS Conference; Vienna, Austria; 18–23 July 2010. Available: http://www.iasociety.org/Abstracts/A200736968.aspx. Accessed 15 October 2013.

[10] Jennings L, Bertrand J, Rech D, Harvey SA, Hatzold K, et al (2014) Quality of voluntary medical male circumcision services during scale-up: a comparative process evaluation in Kenya, South Africa, Tanzania and Zimbabwe. PLoS ONE 9: e79524. 24801073 10.1371/journal.pone.0079524 PMC4011679

[11] Rech D, Spyrelis A, Frade S, Perry L, Farrell M, et al (2014) Implications of the fast-evolving scale-up of adult voluntary medical male circumcision for quality of services in South Africa. PLoS ONE 9: e80577. 24801209 10.1371/journal.pone.0080577 PMC4011681

[12] Rech D, Bertrand JT, Thomas N, Farrell M, Reed J, et al (2014) Surgical efficiencies and quality in the performance of voluntary medical male circumcision procedures in Kenya, South Africa, Tanzania, and Zimbabwe. PLoS ONE 9: e84271. 24802412 10.1371/journal.pone.0084271 PMC4011873

[13] Onyango T (2011) Male Circumcision Consortium, Monitoring and Evaluation Office.

[14] Djimeu Wouabe E (2013) Scoping report on interventions for increasing the demand for voluntary medical male circumcision. Washington (D.C.): International Initiative for Impact Evaluation (3IE).

[15] Macintyre K, Andrinopoulos K, Moses N, Bornstein M, Ochieng A, et al (2014) Attitudes, perceptions and potential uptake of male circumcision among older men in Turkana County, Kenya using qualitative methods. PLoS ONE 9: e83998. 24802112 10.1371/journal.pone.0083998 PMC4011674

[16] Herman-Roloff A, Otieno N, Agot K, Ndinya-Achola J, Bailey RC (2011) Acceptability of medical male circumcision among uncircumcised men in Kenya one year after the launch of the national male circumcision program. PLoS ONE 6: e19814. 21603622 10.1371/journal.pone.0019814 PMC3095626

[17] Westercamp N, Bailey RC (2007) Acceptability of male circumcision for prevention of HIV/AIDS in sub-Saharan Africa: a review. AIDS Behav 11: 341–355. 17053855 10.1007/s10461-006-9169-4 PMC1847541

[18] Gasasira RA, Sarker M, Tsague L, Nsanzimana S, Gwiza A, et al (2012) Determinants of circumcision and willingness to be circumcised by Rwandan men, 2010. BMC Public Health 12: 134. 22340083 10.1186/1471-2458-12-134 PMC3299639

[19] Westercamp M, Agot KE, Ndinya-Achola J, Bailey RC (2012) Circumcision preference among women and uncircumcised men prior to scale-up of male circumcision for HIV prevention in Kisumu, Kenya. AIDS Care 24: 157–166. 21854351 10.1080/09540121.2011.597944 PMC3682798

[20] Plotkin MK, Curran J, Mziray K, Prince H, Mahler J, et al.. (2011) The unpeeled mango: a qualitative assessment of views and preferences of voluntary medical male circumcision in Iringa Region, Tanzania. Dar es Salaam, Tanzania: Jhpiego.

[21] Hatzold K, Mavhu W, Jasi P, Chatora K, Cowan FM, et al (2014) Barriers and motivators to voluntary medical male circumcision uptake among different age groups of men in Zimbabwe: results from a mixed methods study. PLoS ONE 9: e85051. 24802746 10.1371/journal.pone.0085051 PMC4011705

[22] Adamu Ashengo T, Hatzold K, Mahler H, Rock A, Kanagat N, et al (2014) Voluntary medical male circumcision (VMMC) in Tanzania and Zimbabwe: service delivery intensity and modality and their influence on the age of clients. PLoS ONE 9: e83642. 24801882 10.1371/journal.pone.0083642 PMC4011872

[23] Plotkin M, Castor D, Mziray H, Küver J, Mpuya E, et al (2013) “Man, what took you so long?” Social and individual factors affecting adult attendance at voluntary medical male circumcision services in Tanzania. Glob Health Sci Pract 1: 108–116. 25276521 10.9745/GHSP-D-12-00037 PMC4168557

[24] Bollinger L, Adesina A, Forsythe S, Godbole R, Reuben E, et al (2014) Cost drivers for voluntary medical male circumcision using primary source data from sub-Saharan Africa. PLoS ONE 9: e84701. 24802593 10.1371/journal.pone.0084701 PMC4011577

[25] Menon V, Gold E, Godbole R, Castor D, Mahler H, et al (2014) Costs and impacts of scaling up voluntary medical male circumcision in Tanzania. PLoS ONE 9: e83925. 24802022 10.1371/journal.pone.0083925 PMC4011575

[26] Bratt JH, Zyambo Z (2013) Comparing direct costs of facility-based Shang Ring provision versus a standard surgical technique for voluntary medical male circumcision in Zambia. J Acquir Immune Defic Syndr 63: e109–112. 23481667 10.1097/QAI.0b013e31828e9526

[27] Duffy K, Galukande M, Wooding N, Dea M, Coutinho A (2013) Reach and cost-effectiveness of the PrePex device for safe male circumcision in Uganda. PLoS ONE 8: e63134. 23717402 10.1371/journal.pone.0063134 PMC3661578

[28] Njeuhmeli E, Kripke K, Hatzold K, Reed J, Edgil D, et al (2014) Cost analysis of integrating the PrePex medical device into a voluntary medical male circumcision program in Zimbabwe. PLoS ONE 9: e82533. 24801515 10.1371/journal.pone.0082533 PMC4011574

[29] Mavhu W, Frade S, Yongho A, Farrell M, Hatzold K, et al (2014) Provider attitudes toward the voluntary medical male circumcision scale-up in Kenya, South Africa, Tanzania and Zimbabwe. PLoS ONE 9: e82911. 24801632 10.1371/journal.pone.0082911 PMC4011678

[30] Perry L, Rech D, Mavhu W, Frade S, Machaku MD, et al (2014) Work experience, job-fulfillment and burnout among VMMC providers in Kenya, South Africa, Tanzania and Zimbabwe. PLoS ONE 9: e84215. 24802260 10.1371/journal.pone.0084215 PMC4011707

[31] (2008) Avahan—The India AIDS Initiative: The business of HIV prevention at scale. New Delhi: Bill & Melinda Gates Foundation.

[32] Kikaya V, Skolnik L, García MC, Nkonyana J, Curran K, et al (2014) Voluntary medical male circumcision programs can address low HIV testing and counseling usage and ART enrollment among young men: lessons from Lesotho. PLoS ONE 9: e83614. 24801714 10.1371/journal.pone.0083614 PMC4011866

[33] WHO (2010) Considerations for implementing models for optimizing the volume and efficiency of male circumcision services. Field testing edition. Geneva: WHO.

[34] Sgaier SK, Claeson M, Gilks C, Ramesh BM, Ghys PD, et al (2012) Knowing your HIV/AIDS epidemic and tailoring an effective response: how did India do it? Sex Transm Infect 88: 240–249. 22510332 10.1136/sextrans-2011-050382 PMC3351854

[35] Bertrand JT, Rech D, Omondi Aduda D, Frade S, Loolpapit M, et al (2014) Systematic monitoring of voluntary medical male circumcision scale-up: adoption of efficiency elements in Kenya, South Africa, Tanzania, and Zimbabwe. PLoS ONE 9: e82518. 24801374 10.1371/journal.pone.0082518 PMC4011576

[36] (2013) Micro-planning in peer led outreach programs—a handbook. New Delhi: Bill & Melinda Gates Foundation.

[37] Sgaier SK, Ramakrishnan A, Dhingra N, Wadhwani A, Alexander A, et al (2013) How the Avahan HIV prevention program transitioned from the Gates Foundation to the Government of India. Health Affairs 32: 1265–1273. 23836743 10.1377/hlthaff.2012.0646

[38] WHO/AFRO (2013) Progress in scaling up voluntary medical male circumcision for HIV prevention in East and Southern Africa, January – December 2012. Brazzaville: WHO/AFRO.

